# Politics in the State Medical Association

**Published:** 1912-06

**Authors:** 


### POLITICS IN THE STATE MEDICAL ASSOCIATION.

It is much to be regretted that certain indiscreet members of the State Medical Association, at the recent Waco meeting, should have made vigorous attacks on the Governor and the ex-Governor, the former because, under the act of the Legislature creating the two tuberculosis sanatoria, he did not appoint members of the State Association on the Tuberculosis Commission. To this Commission is entrusted the building and inauguration of those State institutions. Instead of doing so, he appointed a Commission composed of laymen (except as to the State Health Officer, who is ex-officio Chairman). These same members censured ex-Governor Campbell because he vetoed the bill creating these sanatoria; and were ready to embrace Governor Colquitt when he approved this bill. Now that he has seen proper to go outside of the State Association for his appointees, the wrath of some is red hot. In the Section on State Medicine a Dr. Shropshire, of Yoakum, fired into Colquitt with ridicule, and he was followed by Dr. O'Farrell, of Richmond, who, however, was called to order. Dr. Steiner, State Health Officer and ex-officio Chairman of the Commission, replied in a forceful and dignified manner, defending the Governor's action. Dr. M. K. Beall, a member of the State Board of Health, and Dr. Clay Johnson, both of Fort Worth, in a published interview in a local paper, denounced the political wrangle as "contemptible and disgusting."

Politics is foreign to the purposes for which the Association was formed and exists—that is, from our standpoint, and is certainly dangerous to its integrity; and, when engaged in, certainly impairs its usefulness and discredits its efforts at medical and sanitary legislation. The people—and the Legislature—witnessing the spleen and disappointment of some members—may well ask, as they already do, *cui bono?* For whose benefit are these new offices created—for the Texas State Medical Association or for the public? Every member who has served on legislative committees knows how difficult it is to make the lawmakers believe that the efforts and activity of the doctors is disinterested; and this sentiment—intensified and emphasized by such evidences of disappointment at not receiving the appointments under recent acts secured by the Association—has always been an obstacle. It is to be hoped that the wise and able and popular physician, Dr. J. S. Turner, President-elect, in his administration, will impress these facts—for facts they are—on the body, and discountenance any future repetition of this disgraceful scene at the Waco meeting.

